# Long‐term follow‐up of task‐specific tremor after thalamotomy: a retrospective observational study

**DOI:** 10.1002/acn3.51953

**Published:** 2023-11-29

**Authors:** Masato Murakami, Shiro Horisawa, Kilsoo Kim, Nobuhiko Takeda, Shinichi Goto, Kotaro Kohara, Takakazu Kawamata, Takaomi Taira

**Affiliations:** ^1^ Department of Neurosurgery, Neurological Institute Tokyo Women's Medical University Tokyo Japan

## Abstract

**Objective:**

This study aimed to report the long‐term results of thalamotomy in 23 patients with task‐specific tremor.

**Methods:**

Data of 23 patients with task‐specific tremor who underwent ventralis intermedius nucleus and posterior part of ventro‐oral nucleus thalamotomy at the Tokyo Women's Medical University Hospital between 2010 and 2022 were retrospectively analyzed. To evaluate neurological conditions, the severity of task‐specific tremor was divided into 0 (no tremor), 1 (slightly tremulous), 2 (moderately tremulous), 3 (accomplishing tasks with great difficulty), and 4 (unable to complete tasks). We also used the subscores “handwriting” (0–4) and “spiral drawing” (0–4) of the Clinical Rating Scales for Tremor. Evaluation scales were presented as medians and interquartile ranges.

**Results:**

The severities of task‐specific tremor were 3.0 (3.0–4.0) preoperatively and 0.0 (0.0–0.0, *p* < 0.0001) at the last available evaluation. The writing and spiral drawing of the Clinical Rating Scales for Tremor significantly improved from 3.0 (3.0–4.0) and 3.0 (2.0–3.0) preoperatively, respectively, to 0.0 (0.0–0.0, *p* < 0.0001) and 0.0 (0.0–0.0, *p* < 0.0001) at the last available evaluation, respectively. The mean clinical follow‐up period was 62.7 ± 26.0 months. Seven (30.4%) patients had focal hand dystonia, which newly developed on the ipsilateral side of the tremor at 2–45 months after the surgery. No serious complications were observed.

**Interpretation:**

Thalamotomy significantly improves task‐specific tremor with high long‐term efficacy, and long‐term follow‐up is important because focal hand dystonia can develop postoperatively.

## Introduction

Task‐specific tremor (TST) is an action tremor that develops only or mainly when performing specific tasks.^1^ The specific tasks include writing, playing instruments, and using drills and scissors.^2^, ^3^, ^4^ Primary writing tremor (PWT) is a representative form of TST in which tremor predominantly occurs and interferes with handwriting.^5^ For decades, it has been debated whether PWT is a form of dystonic tremor, a variant of essential tremor, or a separate entity.^6^, ^7^ The etiology of TST remains controversial, and the treatments include oral medications, botulinum toxin injections, and stereotactic neurosurgery of the ventralis intermedius nucleus (Vim) by lesioning (thalamotomy) and electrical stimulation (deep brain stimulation [DBS]).^8^, ^9^, ^10^, ^11^ For medically refractory TST, stereotactic Vim thalamotomy and DBS are highly effective. However, to date, only seven studies including 13 patients examining the surgical treatment of TST have been reported (3 patients underwent thalamotomy, 9 underwent DBS, and 1 underwent focused ultrasound [FUS] thalamotomy).^8^, ^9^, ^10^, ^11^, ^12^, ^13^, ^14^ Considering the paucity of data, we aimed to report long‐term results of thalamotomy in 23 patients with TST. This is the largest patient population and longest follow‐up study to describe outcomes in patients with TST who underwent neurosurgical treatments.

## Patients and Methods

### Subjects

Data from 23 patients with TST who underwent thalamotomy at the Tokyo Women's Medical University Hospital between 2010 and 2022 were retrospectively collected and analyzed. All patients had medically refractory TST. DBS was rejected owing to refusal to have a mechanical device implanted and difficulty accessing hospitals that manage DBS from remote areas. This study was approved by the Institutional Review Board of Tokyo Women's Medical University (No. 3576). The data for this study were retrospectively collected and analyzed. Considering the observational nature of the study, patient consent was waived.

### Surgical procedures

**Figure 1:**
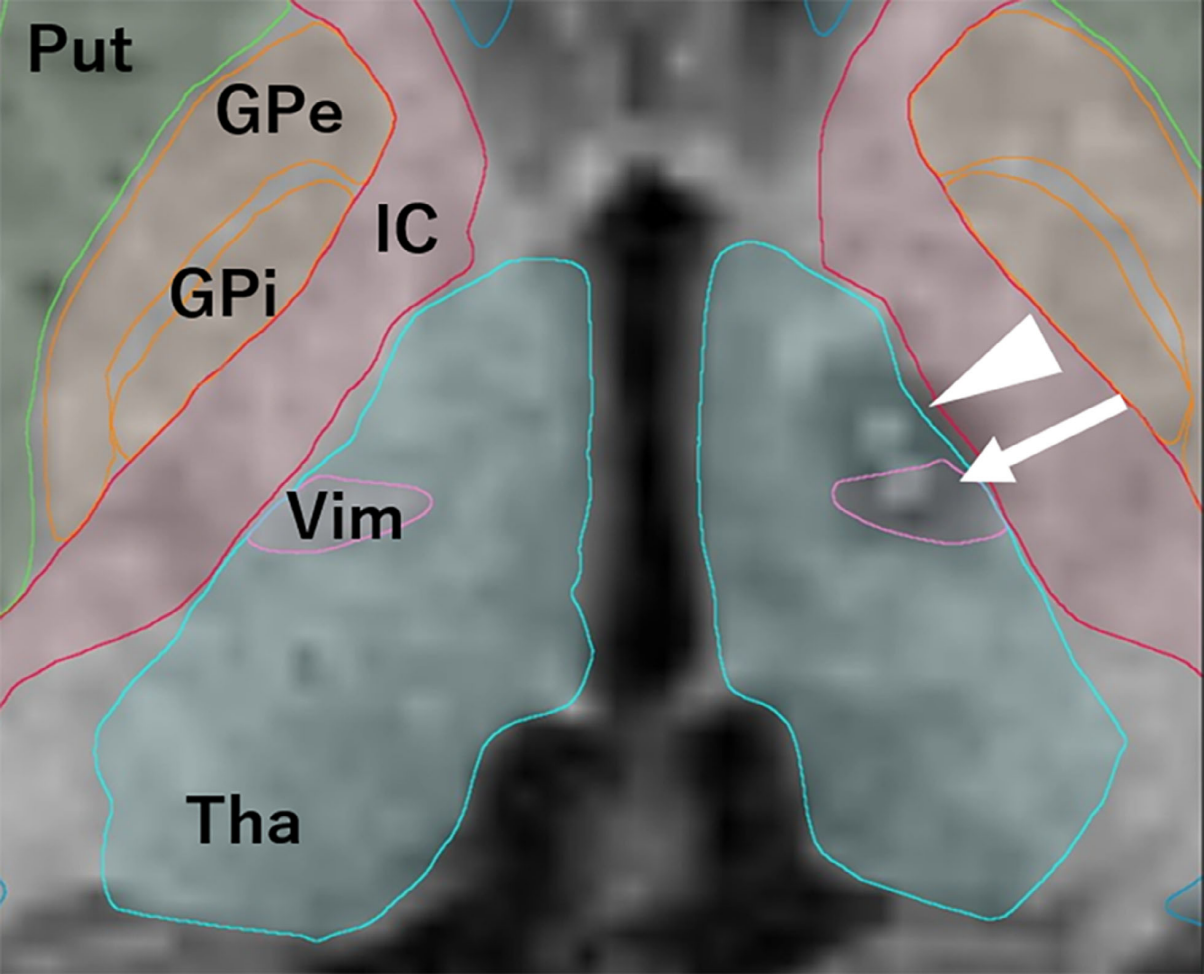
Post‐Vop‐Vim thalamotomy MRI. Radiofrequency lesions at the Vop (arrowhead) and Vim (arrow) were confirmed using the Brainlab Elements anatomical mapping tool. Thalamus (blue), Vim nucleus (pink), putamen (green), globus pallidus internus (Gpi, orange) and externus (Gpe, orange), and internal capsule (red). MRI, magnetic resonance imaging; Vim, ventral intermediate nucleus; Vop, posterior part of the ventro‐oral nucleus.

Stereotactic planning was performed using the Leksell SurgiPlan (Elekta, Stockholm, Sweden) and BrainLab Elements (BrainLab, Munich, Germany). Stereotactic target of the Vim nucleus of the thalamus was set 12.5–15 mm lateral from the midline or 11–12 mm lateral from the third ventricle wall, 4–6 mm anterior from the posterior commissure (PC), and on the anterior commissure (AC)–PC (AC–PC) plane. The surgery was performed under local anesthesia. Microelectrode recordings were not used. We used a monopolar radiofrequency probe (1.0‐mm‐diameter tip with 4.0 mm uninsulated length) and Leksell Neuro Generator (Elekta) for macrostimulation (130 Hz, 100‐μs pulse width, up to 4.3 mA) and coagulation. Coagulation was performed at 70°C for 40 s at the tentative target, and the electrode was withdrawn in 3‐mm increments to increase the lesion size, resulting in two contiguous lesions. We also added similar lesions 3 mm anteromedial to the target, which corresponded to the posterior part of the ventro‐oral (Vo) nucleus (Vop). Before coagulation, we performed macrostimulation to carefully assess for tremor improvement and side effects (sensory and capsular responses). Within 24 h of surgery, a postoperative magnetic resonance imaging (MRI) was performed to confirm the location of the lesion (Fig. 1).

### Evaluation procedures

To evaluate neurological conditions, TST severity was divided into 0 (no tremor), 1 (slightly tremulous), 2 (moderately tremulous), 3 (accomplishing tasks with great difficulty), and 4 (unable to complete tasks). This rating scale was used to assess tremor induced by specific task at the onset of TST. We also used the subscores “handwriting” and “spiral drawing” of the Clinical Rating Scales for Tremor (CRST) in our evaluation.^15^ Handwriting consists of 0–4 points (0 = normal, 1 = mildly normal, slightly untidy, tremulous, 2 = moderately abnormal, legible, but with considerable tremor, 3 = markedly abnormal, illegible, and 4 = severely abnormal, unable to keep pencil or pen on paper without holding hand down with the other hand). Spiral drawing consists of 0–4 points (0 = normal, 1 = slightly tremulous, may cross lines occasionally, 2 = moderately tremulous or cross lines frequently, 3 = accomplishes tasks with great difficulty, many errors, and 4 = unable to complete drawing). These rating scales were presented as medians and interquartile ranges. Clinical follow‐up with tremor evaluations and surgical complications was performed before surgery, at 1 week and 3 months after surgery, and at each available final follow‐up period. Postoperative images were validated using Brainlab Elements, and the analyses included the location (coordinates) and volume of the lesions.

### Statistical analyses

The Wilcoxon signed‐rank test was used to compare pre‐ and postoperative tremors (at the final follow‐up) in writing, spiral drawing, and TST severity. All statistical analyses were performed using the JMP statistical package, version 15.0.0 (SAS Institute Inc., Cary, North, USA). All statistical tests were two‐tailed, and significance was set at *p* < 0.05.

## Results

Table 1 shows the patients' demographic characteristics. Detailed individual patient's data are shown in Table S1. In total, 23 (male, 19; female, 4) patients were included in this study. The mean age at dystonia onset and surgery were 40.5 ± 15.0 and 54.0 ± 10.3 years, respectively. The mean clinical follow‐up period was 62.7 ± 26.0 months. All surgeries were performed on the left side. Tremor‐inducing tasks at the onset of TST included writing in 15 patients, dental drilling in 3 patients, ophthalmic instruments in 1 patient, cooking movement in 1 patient, tattooing in 1 patient, keyboard typing in 1 patient, and tennis movement in 1 patient. Of the 8 patients who did not have writing tremor at the onset of TST, 7 developed writing tremor later. One patient had a cooking movement‐specific tremor but did not have writing‐induced tremor.

**Table 1:** Patient characteristics.

Number of patients	23
Sex	Male: 19, Female: 4
Age at onset (year)	40.5 ± 15.0
Age at surgery (year)	54.4 ± 11.7
Dominant hand	Right: 23
Side of surgery	Left: 23
Follow‐up period (month)	62.7 ± 26.0
Occupation	
Office worker	14
Dentist	3
Calligrapher	2
Ophthalmologist	1
Tattoo artist	1
Cooker	1
Lawyer	1

All evaluation scale scores significantly decreased from the preoperative period to the last available evaluation (Table 2). TST severity (23 patients) was 3.0 (3.0–4.0) preoperatively, which significantly decreased to 0.0 (0.0–0.0, *p* < 0.0001) at the last available evaluation. One patient did not have a writing tremor and was therefore not included in the calculation of the CRST assessments. The writing (22 patients) and spiral drawing (22 patients) of the CRST significantly improved from 3.0 (3.0–4.0) and 3.0 (2.0–3.0) preoperatively, respectively, to 0.0 (0.0–0.0, *p* < 0.0001) and 0.0 (0.0–0.0, *p* < 0.0001) at the last available evaluation, respectively. A representative case is shown in Video S1. Detailed individual clinical outcomes are shown in Table S2.

**Table 2:** Clinical outcomes of clinical rating scale for tremor.

	Pre	1 week	3 months	Last^a^	
Writing	3.0 (3.0–4.0)	0.0 (0.0–0.0)	0.0 (0.0–0.0)	0.0 (0.0–0.0)	*p* < 0.0001^b^
Spiral	3.0 (2.0–3.0)	0.0 (0.0–0.0)	0.0 (0.0–0.0)	0.0 (0.0–0.0)	*p* < 0.0001^b^
Tasks	3.0 (3.0–4.0)	0.0 (0.0–0.0)	0.0 (0.0–0.0)	0.0 (0.0–0.0)	*p* < 0.0001^b^

Data presented as median (interquartile range). Each scores evaluates the severity of tremor (range: 0–4, higher scores indicate greater severity).

^a^ The mean last available follow‐up period: 62.7 ± 26.0 months.

^b^ By comparison of preoperative and the last available follow‐up evaluations.

**Figure 2:**
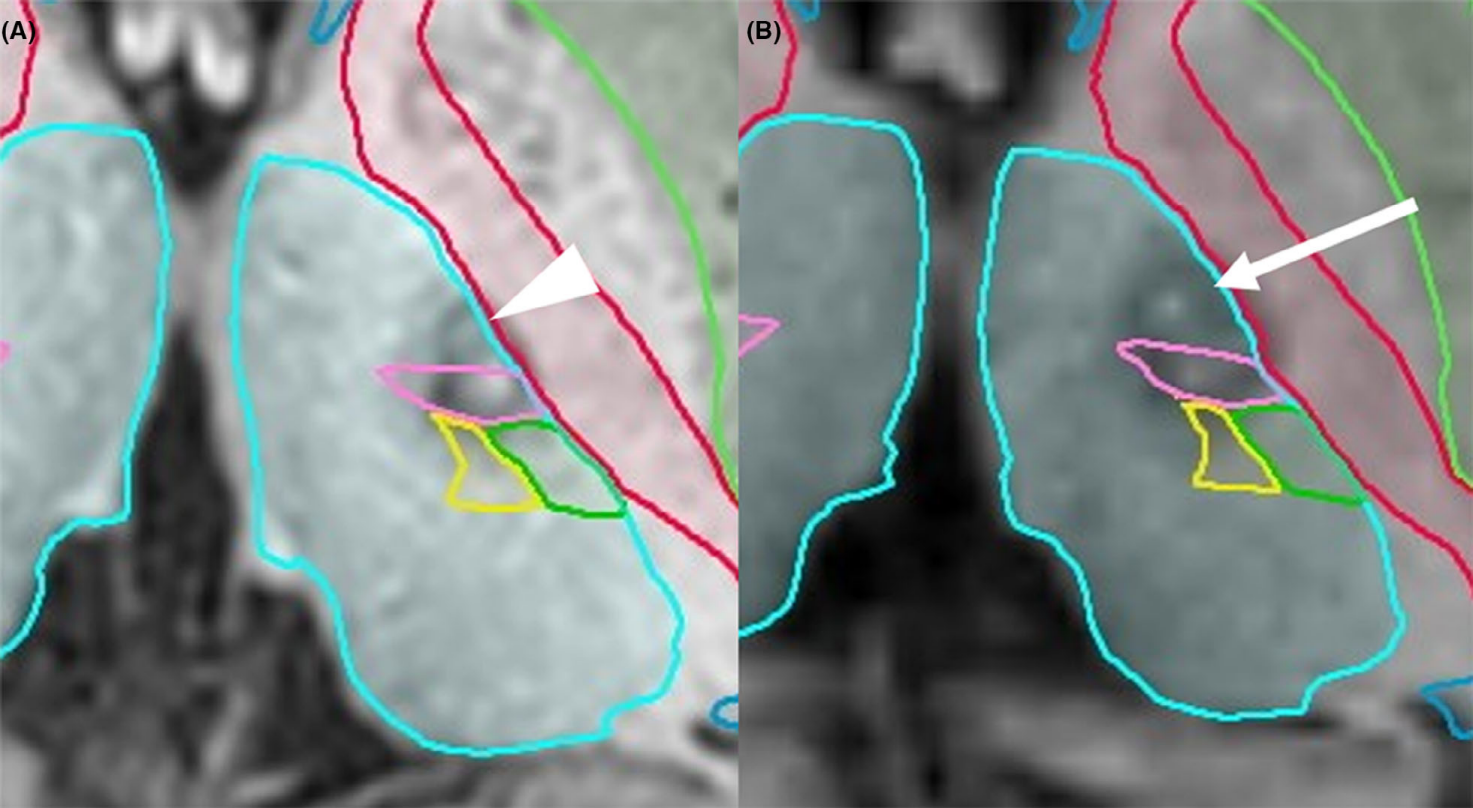
Postoperative MRI images in a patient with focal hand dystonia developed after thalamotomy. Task‐specific tremor was well controlled by the first thalamotomy (A). Four months after the first thalamotomy, focal hand dystonia developed. The second thalamotomy resulted in complete resolution of the focal hand dystonia (B). The Vop lesion in the first thalamotomy (arrowhead) is considerably smaller than that in the second thalamotomy (arrow). MRI, magnetic resonance imaging.

Adverse events are shown in Table 3. The most common adverse event was temporary treated side hypotonia. Among the patients, 13 experienced temporary or prolonged adverse events, which included tongue numbness in 1 (8.7%) patient, dysarthria in 2 (8.7%), and dysgeusia in 1 (4.3%). No serious adverse events that required prolonged hospitalization were observed. Seven (30.4%) patients had focal hand dystonia (FHD) that developed on the same side of the tremor at 2–45 months after the surgery. Among them, 1 underwent another ventralis oralis posterior (Vop)‐Vim thalamotomy for FHD (right wrist flexion) 15 months after the first thalamotomy (Fig. 2, Videos S2 and S3). The second thalamotomy improved the patient's right FHD, which did not recur 20 months after the second thalamotomy (Video S3). Two patients experienced recurrent tremors and underwent revision surgery. One of them developed dystonia in the hand on the same side as the tremor 2 months after reoperation. Additionally, 2 patients developed tremor in the contralateral hand. During the follow‐up period, we confirmed that there was no newly developed possible parkinsonism, including resting tremor, dystonia other than the FHD of the tremor side, postural instability, and gait disturbance. A detailed analysis of the lesion location and volume is shown in Table 4. The coordinate of the center of the lesion was 14.4 mm lateral from the midline (11.4 mm lateral from the third ventricle wall), 5.9 mm anterior from the PC, and 1.0 mm inferior to the AC–PC plane.

**Table 3:** Adverse events.

Temporary	
Limb hypotonia	5
Tongue numbness	2
Dysarthria	2
Hemispatial neglect	1
Prolonged	
Tongue numbness	2
Dysarthria	2
Dysgeusia	1

**Table 4:** Lesion analysis.

Mediolateral plane, mm	14.4 ± 1.0
From third ventricle wall, mm	11.4 ± 1.3
Anteroposterior plane, mm^a^	5.9 ± 1.3
Dorsoventral plane, mm	−0.9 ± 0.8
Volume of lesion, mm^3^	81.9 ± 25.4

^a^ Distance measured from posterior commissure.

## Discussion

In this study, we reported long‐term results of Vim‐Vop thalamotomy performed on 23 patients with TST. We found that at a mean follow‐up period of 62.7 months, 21 patients had long‐term resolution of TST, and 2 had residual tremor. Seven patients developed dystonia in the upper extremity of the treated hand. Thus, this study showed that thalamotomy improved TST in the long term.

Reports on the surgical treatment of TST are extremely limited. To the best of our knowledge, only 13 patients have been reported to date. Of these, 3 included patients treated with radiofrequency (RF), 9 with DBS, and 1 with FUS. Four reports using objectively available scores reported good improvements on the CRST scale scores: 85.2%, 85.7%, 93.3%, and 94.4%.^10^, ^11^, ^12^, ^13^ Other studies also reported good response in 4 and 5 patients evaluated using the description of tremor resolution.^9^, ^14^ However, no reports are available on detailed long‐term follow‐up of TSTs. Ohye et al. reported a case with complete cure for TST at a follow‐up period of 30 months after surgery.^9^ Other reports included two cases with 6‐month postoperative follow‐up period and two cases with 12‐month postoperative follow‐up period,^11^, ^12^, ^13^, ^14^ whereas the other reports did not specify the follow‐up period. In our study, all 3 patients followed up for >10 years after surgery had complete resolution of tremor, and 7 of the 8 patients followed up for >5 years after surgery had complete resolution of tremor. Considering these results, TST improves significantly well with thalamic surgery in the long term.

To date, the pathogenesis of TST remains unclear. TST has been suggested as a subtype of essential or dystonic tremor.^4^, ^5^, ^6^, ^7^ Notably, our study showed that 7 (30.4%) patients presented with FHD after surgery for TST, with the FHD onset between 2 and 45 months after surgery. TST patients usually do not develop dystonia in the long‐term course of the disease. We may have missed the subtle dystonic features at preoperative evaluations in 7 patients with FHD onset after the surgery, suggesting that preexisting dystonic features became more prominent with the tremor arrest after the surgery. Another possibility of FHD development in this study is that FHD may have been caused by thalamotomy. The development or worsening of dystonia after thalamic surgery for tremor has only been reported in a small number of cases.^16^, ^17^ Picillo et al. reported dystonia in the upper or lower extremity on the treated side after Vim‐DBS or RF‐Vim thalamotomy in 6 patients with tremor symptoms.^17^ Among these cases, the upper extremity dystonia developed 3 months after Vim‐thalamotomy for tremor in tremor‐dominant PD. Stimulation adjustments, globus pallidus internus (GPi)‐DBS, and botulinum toxin injections were administered to treat upper extremity dystonia after thalamic surgery. However, these were only partially effective. Martino et al. reported that two patients with tremor and dystonia had worsened preexisting hand dystonia and newly developed upper arm dystonia after FUS Vim thalamotomy^16^; however, the treatment for the two patients' was not described. Of the 7 patients who developed dystonia in our study, 1 underwent Vo thalamotomy, which resulted in complete resolution of the hand dystonia. Analysis of postoperative MRI images showed that the lesion in the first tremor surgery was centered on the Vim nucleus (Fig. 2A). During the second surgery, the lesion covered the posterior component of the Vo nucleus (Fig. 2B). The thalamic Vo nucleus is an effective target for upper extremity dystonia.^18^ By contrast, GPi has limited efficacy in upper extremity dystonia. Fukaya et al. reported that in a patient with writer's cramp in whom two DBS leads were implanted (GPi and Vo/VIM), the clinical effect of thalamic stimulation was better than that of pallidal stimulation.^19^ The limited improvement of GPi‐DBS in upper limb dystonia in the study by Picillo et al. may be attributable to the treatment targeting the GPi.

We believe that the optimal therapeutic target for TST is not only the Vim nucleus but also the Vop nucleus. The Vim nucleus receives input from the cerebellum, whereas the Vop receives input from the globus pallidus.^20^ During the long‐term follow‐up period in our study, dystonia at the site of tremor onset was observed in 7 of 23 patients, suggesting that a dystonic component is involved in TST pathogenesis. Although the Vo nucleus is the optimal therapeutic target for FHD, our recent experience suggests that the Vop region is the most effective site for treating FHD. Racette et al. reported a 94.4% improvement of TST after Vim‐DBS for PWT.^10^ The electrode is located 14 mm lateral and 0.25 mm posterior to the midpoint of AC–PC.^10^ This is the same position of the Vo nucleus that we usually use for FHD and corresponds to the VLa nucleus in the Morel atlas.^21^ Meng et al. also reported TST treatment using FUS thalamotomy.^13^ The location of the target (Vim nucleus) was 14.3 mm from the midline and 6.4 mm anterior to the PC.^13^ Vim lesion using FUS resulted in moderate improvement and the appearance of abnormal tongue sensation. Additional FUS lesioning of the Vop, 3 mm anterior to the treated target, resulted in a significant improvement in TST (93.3% improvement). We also treated TST in the same manner as Meng et al. and achieved similar high therapeutic efficacy and long‐term improvement. However, our method of targeting Vim‐Vop may not adequately cover the entire Vop nucleus. In our study, the patient who developed FHD after the first surgery and underwent additional Vop‐Vim thalamotomy, coagulation of the Vop nucleus by the initial procedure was considered insufficient. When the posterior part of the Vim nucleus is targeted, an area 3 mm anterior to the target is often located at the boundary between the Vop and Vim. Radiofrequency ablation using our 1‐mm‐diameter electrode at the boundary region between the Vop and Vim is thought to result in insufficient ablation of the anterior component of the nucleus Vop. In the second surgery, radiofrequency ablation was performed mainly on the Vop nucleus, and the FHD significantly improved during the surgery. Postoperative MRI revealed a distinct lesion in the Vop nucleus (anterior to Vim). Therefore, if the Vim and Vop nuclei can be accurately treated, dystonia development after thalamic surgery in TST may also be reduced, although whether thalamic surgery induces dystonia or causes unmasked dystonia remains unclear. Thalamic procedures can disrupt either or both the cerebello‐thalamic and cortico‐striato‐pallido‐thalamo‐cortical loops, resulting in an altered thalamocortical outflow and causing movement disorders.^17^, ^22^ From this perspective, it seems reasonable to simultaneously treat Vim, which belongs to the cerebellar pathway, and Vop, which belongs to the pallidal pathway.

This study had some limitations. Guidelines for diagnosing TST conclusively have not yet been established, and the patients in this study may include patients with essential tremor or dystonic tremor. This was an open‐label study that had observer and patient biases. Subtle neurological deficits and dystonic or parkinsonian features may have also been overlooked owing to the lack of pre‐ and postoperative unified evaluation procedures.

In conclusion, this study suggested that long‐term outcomes of TST can be improved using thalamic surgery. Furthermore, long‐term follow‐up is important because FHD can develop postoperatively.

## Author Contributions

MM: analysis and acquisition of data and writing the manuscript; SH: study design, patient selection, analysis of imaging data, interpretation and acquisition of data, and writing and revising the manuscript; KK: acquisition of data; NT: acquisition of data; KK: acquisition of data; TK: study design; TT: study design, patient selection, acquisition, and interpretation of data.

## Funding Information

This study was funded by the Japan Brain Foundation and the Japan Society for the Promotion of Science KAKENHI (Grant JP21K09113).

## Conflict of Interest

The authors report no conflicts of interest related to the research covered in this study.

## Supporting information


**Table S1.** Individual patient's demographics.

**Table S2.** Individual clinical outcomes.

**Video S1.** Pre‐ and postoperative writing of a representative successful case.

**Video S2.** Pre‐ and intraoperative conditions in a patient with later development of focal hand dystonia.

**Video S3.** Pre‐, intra‐, and postoperative conditions of focal hand dystonia.

## Data Availability

The data in this study are available upon request from qualified investigators.
