# Supplementary material for: Long‐term follow‐up of task‐specific tremor after thalamotomy: a retrospective observational study
Source: Ann Clin Transl Neurol. 2023 Nov 29;11(2):321–7. doi: 10.1002/acn3.51953 (PMC10863909; doi:10.1002/acn3.51953)
Supplement: Supplementary file 1 — Table S1. Individual patient's demographics. [file ACN3-11-321-s002.docx]

Supplementary table1. Individual patient’s demographics

|  |  |  |  |  |  | Before surgery | | | |
| --- | --- | --- | --- | --- | --- | --- | --- | --- | --- |
| Case | Sex | Age at onset  (year) | Age at surgery  (year) | Tasks at onset | Other affected tasks | Tasks | Writing | Spiral | Posture** |
| 1 | F | 35 | 43 | Writing | Chopsticks | 3 | 3 | 3 | 1 |
| 2 | M | 45 | 50 | Writing |  | 3 | 3 | 2 | 0 |
| 3 | F | 20 | 36 | Dental drilling | Writing, Chopsticks | 4 | 4 | 3 | 0 |
| 4 | M | 20 | 47 | Writing |  | 3 | 3 | 3 | 0 |
| 5 | M | 27 | 40 | Writing |  | 2 | 2 | 2 | 1 |
| 6 | M | 62 | 68 | Writing |  | 4 | 4 | 3 | 1 |
| 7 | M | 64 | 72 | Writing |  | 4 | 4 | 2 | 0 |
| 8 | M | 59 | 65 | Writing | Chopsticks | 2 | 2 | 2 | 1 |
| 9 | F | 10 | 46 | Writing |  | 3 | 3 | 3 | 2 |
| 10 | M | 50 | 60 | Writing |  | 3 | 3 | 0 | 0 |
| 11 | M | 56 | 66 | Dental drilling | Writing, Chopsticks | 3 | 3 | 3 | 1 |
| 12 | M | 34 | 37 | Dental drilling | Writing, Toothbrash | 3 | 3 | 3 | 0 |
| 13 | M | 47 | 57 | Writing |  | 4 | 4 | 4 | 1 |
| 14 | M | 40 | 43 | Writing |  | 4 | 4 | 2 | 0 |
| 15 | F | 51 | 54 | Writing |  | 3 | 3 | 3 | 0 |
| 16 | M | 40 | 64 | Ophthalmic instruments | Writing | 4 | 4 | 4 | 0 |
| 17 | M | 29 | 38 | Tattooing | Writing | 3 | 3 | 2 | 0 |
| 18 | M | 50 | 53 | Cooking movements |  | 3 | 0* | 0* | 0* |
| 19 | M | 31 | 41 | Writing |  | 4 | 4 | 3 | 0 |
| 20 | M | 40 | 65 | Writing |  | 4 | 4 | 2 | 0 |
| 21 | M | 60 | 65 | Keyborad typing | Writing, Chopsticks | 4 | 4 | 3 | 0 |
| 22 | M | 22 | 52 | Tennis movements | Writing | 2 | 2 | 2 | 0 |
| 23 | M | 40 | 53 | Writing |  | 3 | 3 | 3 | 1 |

*This patient did not have a writing tremor and was therefore not included in the calculation of the writing and spiral drawing of the clinical rating scales for tremor.

**Postural tremor consists of 0–4 points (0 = none, 1 = slight (amplitude < 0.5cm). May be intermittent, 2 = Moderate amplitude (0.5-1.0 cm). May be intermittent, 3 = Marked amplitude (1-2 cm), and 4 = Severe amplitude (> 2cm)).
